# Mitochondrial p38 Mitogen-Activated Protein Kinase: Insights into Its Regulation of and Role in LONP1-Deficient Nematodes

**DOI:** 10.3390/ijms242417209

**Published:** 2023-12-07

**Authors:** Eirini Taouktsi, Eleni Kyriakou, Evangelia Voulgaraki, Dimitris Verganelakis, Stefania Krokou, Stamatis Rigas, Gerassimos E. Voutsinas, Popi Syntichaki

**Affiliations:** 1 Laboratory of Molecular Genetics of Aging, Biomedical Research Foundation of the Academy of Athens, Center of Basic Research, 11527 Athens, Greece; eirinitaouktsi@gmail.com (E.T.); ekyriakou@biol.uoa.gr (E.K.); evageliavoulgaraki123@gmail.com (E.V.); dimitrisvrgnlks@gmail.com (D.V.); krokoustefania@gmail.com (S.K.); 2 Department of Biotechnology, Agricultural University of Athens, 11855 Athens, Greece; srigas@aua.gr; 3 Department of Biological Applications & Technology, University of Ioannina, 45500 Ioannina, Greece; 4 Laboratory of Molecular Carcinogenesis and Rare Disease Genetics, Institute of Biosciences and Applications, National Center for Scientific Research “Demokritos”, Aghia Paraskevi Attikis, 15341 Athens, Greece; mvoutsin@bio.demokritos.gr

**Keywords:** mitochondria, ageing, heat stress response, *C. elegans*, LONP-1, PMK-3, ZIP-2

## Abstract

p38 Mitogen-Activated Protein Kinase (MAPK) cascades are central regulators of numerous physiological cellular processes, including stress response signaling. In *C. elegans*, mitochondrial dysfunction activates a PMK-3/p38 MAPK signaling pathway (MAPK^mt^), but its functional role still remains elusive. Here, we demonstrate the induction of MAPK^mt^ in worms deficient in the *lonp-1* gene, which encodes the worm ortholog of mammalian mitochondrial LonP1. This induction is subjected to negative regulation by the ATFS-1 transcription factor through the CREB-binding protein (CBP) ortholog CBP-3, indicating an interplay between both activated MAPK^mt^ and mitochondrial Unfolded Protein Response (UPR^mt^) surveillance pathways. Our results also reveal a genetic interaction in *lonp-1* mutants between PMK-3 kinase and the ZIP-2 transcription factor. ZIP-2 has an established role in innate immunity but can also modulate the lifespan by maintaining mitochondrial homeostasis during ageing. We show that in *lonp-1* animals, ZIP-2 is activated in a PMK-3-dependent manner but does not confer increased survival to pathogenic bacteria. However, deletion of *zip-2* or *pmk-3* shortens the lifespan of *lonp-1* mutants, suggesting a possible crosstalk under conditions of mitochondrial perturbation that influences the ageing process. Furthermore, loss of *pmk-3* specifically diminished the extreme heat tolerance of *lonp-1* worms, highlighting the crucial role of PMK-3 in the heat shock response upon mitochondrial LONP-1 inactivation.

## 1. Introduction

The evolutionarily conserved p38 mitogen-activated protein kinase (p38 MAPK) signaling cascade transmits diverse extracellular cues to intracellular targets, regulating mainly stress responses, but also other vital cellular processes, such as cell proliferation, differentiation, immune responses, tumorigenesis and apoptosis [1,2]. The *C. elegans* genome contains three p38 MAPK homologs, PMK-1, PMK-2 and PMK-3, which are encoded by one polycistronic transcript of an operon under the activity of a broadly expressed promoter [3]. PMK-1 and PMK-2 function redundantly in the nervous system during development [4]. The neuronal functions of PMK-1 and PMK-2 are regulated by a signaling cascade consisting of the adaptor protein TIR-1, which is orthologous to mammalian Sterile α and Armadillo Motif (SARM), and the upstream kinases NSY-1 (MAP3K) and SEK-1 (MAPKK), which are orthologous to mammalian Apoptosis Signal-Regulating Kinase 1 (ASK1) and Mitogen-Activated Protein Kinase Kinase 3/6 (MKK3/6), respectively [5]. In contrast, PMK-3 and its upstream kinases DLK-1 (MAP3K) and MKK-4 or MLK-1(MAPKKs) act in a cascade that regulates synapse formation [6] or axon regrowth after injury [7,8]. PMK-3 also promotes the differentiation of chemosensory BAG neurons, which sense microbe-derived carbon dioxide (CO_2_) in worms [9,10].

Several studies in *C. elegans* have uncovered additional roles of PMK-1/p38 MAPK in environmental and cellular stress responses. For example, the PMK-1 pathway has a key role in regulating the expression of innate immune genes in response to various pathogens [11,12]. PMK-1 is the sole effector of the TIR-1/NSY-1/SEK-1 cascade, functioning in the intestines of worms to promote host pathogen resistance [13,14,15,16]. The PMK-1 signaling pathway is also activated in response to abiotic stressors such as cadmium [17,18] and cholesterol deprivation [19]. Additionally, during oxidative stress, it acts in the intestine to phosphorylate SKN-1, the worm ortholog of mammalian Nuclear Factor-Erythroid 2-Related Factor (NRF) transcription factors, mainly regulating transcription of phase II detoxification genes [20,21,22]. Furthermore, PMK-1 and SKN-1 activities have been linked to the heat tolerance of *C. elegans*, as heat stress induces nuclear translocation of PMK-1 in intestinal cells and depletion of *pmk-1* reduces the survival rate of worms at high temperatures [23].

In contrast to PMK-1, there is little knowledge about the role of the PMK-3 signaling pathway in stress responses. A mitochondrial MAPK (MAPK^mt^) cascade defined by DLK-1 (MAP3K), SEK-3 (MAPKK) and PMK-3 kinases was identified to be required for lifespan extension of *C. elegans* mutants with disrupted mitochondrial activity [24]. It seems that the PMK-3 pathway defines another cellular retrograde response that is activated in complement to the mitochondrial unfolded protein response (UPR^mt^), a conserved surveillance mechanism by which metazoans respond to mitochondrial stress [25]. During UPR^mt^ activation, an increased unfolded/misfolded protein load inside the mitochondria redirects the basic leucine zipper (bZIP) transcription factor ATFS-1 from the matrix to the nucleus, regulating the expression of both mitochondrial protective genes and metabolism-related genes [25,26]. In the absence of mitochondrial stress, ATFS-1 is trafficked to the mitochondrial matrix and rapidly degraded by the ATP-dependent protease LONP-1, the ortholog of mammalian LonP1 [25].

LonP1 protease belongs to the ATPase associated with diverse cellular activities (AAA^+^) family with major roles in mitochondrial protein quality control [27,28,29]. We have previously shown that deletion of the *lonp-1* gene in *C. elegans* increases endogenous levels of ROS, impacts the mitochondrial function and impairs physiological processes, such as growth, behavior and lifespan [30]. Loss of LONP-1 induces the UPR^mt^, while the accompanied activation of several cytoplasmic UPR (UPR^cyt^) responses was shown to increase the resistance of mutants to heat and other forms of stress. In addition to worms, genetic or pharmacological inhibition of human LonP1 in cancer cells induces similar mitochondrial and cytoplasmic UPR mechanisms [30]. Although the induction of such UPR pathways could result in lifespan extension of *C. elegans*, the short-lived phenotype of the *lonp-1* mutants implies the existence of additional factors/mechanisms that modulate their lifespan and possibly their viability under adverse environmental conditions. In this study, we demonstrated the induction of the MAPK^mt^ signaling pathway in *lonp-1* mutants and we examined its relationship with other mitochondrial surveillance mechanisms, in addition to the effects of its disruption on physiological functions of *lonp-1*-deficient animals. The described relationships and effects might be evolutionarily conserved, thus shedding some light on human health and mitochondrial dysfunction-related diseases.

## 2. Results

### 2.1. The MAPK^mt^ Pathway Is Active in Lonp-1-Deficient Mutants

**Figure 1:**
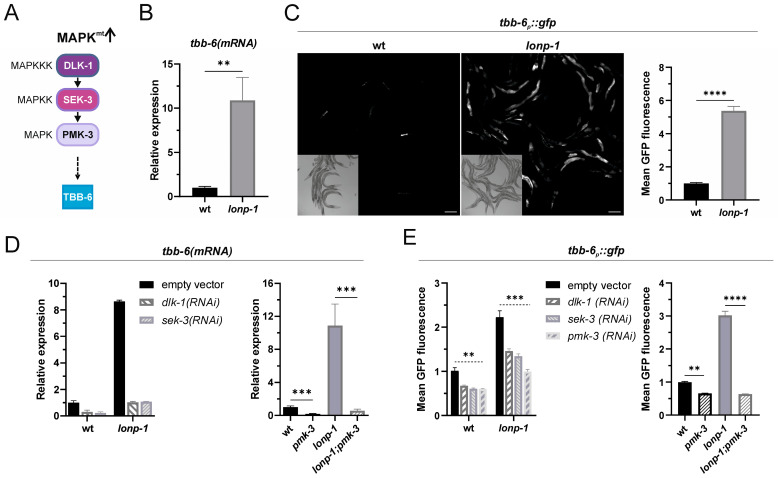
Activation of the mitochondrial MAPK (MAPK^mt^) signaling pathway in *lonp-1*-deficient worms. (**A**) Schematic representation of the MAPK^mt^ pathway consisting of the DLK-1/SEK-3/PMK-3 kinase cascade and the downstream target gene, *tbb-6*. (**B**) Relative mRNA quantification of endogenous *tbb-6* gene expression levels in wt and *lonp-1*-knockout (ko) mutants. (**C**) Representative microscopy images and GFP fluorescence quantification of the *tbb-6_p_::gfp* reporter in wt and *lonp-1(ko)* worms. (**D**,**E**) Quantification of endogenous *tbb-6* gene expression or the *tbb-6_p_::gfp* reporter in wt and *lonp-1(ko)* mutants upon disruption of the MAPK^mt^ signaling components. Experiments were performed on 1-day-old adult worms grown on NGM plates, seeded with *E. coli* OP50 or with *E. coli* HT115 bacteria transformed with the indicated RNAi construct (and empty vector as a control) at 20 °C. Scale bar indicates 100 µm. Values are presented as means ± SEM, and asterisks denote statistical significance assessed with an unpaired Student’s *t*-test or with a two-way ANOVA followed by a post hoc Tukey’s test, ** *p* ≤ 0.01, *** *p* ≤ 0.001, **** *p* ≤ 0.0001.

Mitochondrial dysfunction caused by disruption of the mitochondrial electron transport chain, such as mutations in the Rieske iron-sulfur polypeptide 1 (*isp-1*) of complex III, has previously been shown to activate the MAPK^mt^ pathway, which consists of DLK-1, SEK-3, and PMK-3 kinases and induces transcription of the *tbb-6* gene [24] (Figure 1A).

*tbb-6* encodes for a structurally diverged member of the beta-tubulin family in *C. elegans* [31], with no reported specific functions or overt mutant phenotypes. We examined the expression levels of *tbb-6* in synchronized young adults of wild-type (wt) and *lonp-1* knockout strains and we observed elevated mRNA levels of the endogenous *tbb-6* gene (Figure 1B), as well as a strong induction of a transcriptional reporter *tbb-6_p_::gfp* (Figure 1C) in the intestine and pharyngeal muscles of *lonp-1* compared to wt animals. Mild exposure to *isp-1(RNAi)* further increased the fluorescence of the reporter in *lonp-1*, indicating a strong additive mitochondrial dysfunction in *lonp-1;isp-1(RNAi)* adults (interaction *p* < 0.0005 in a two-way ANOVA; Figure S1A). In addition to *tbb-6*, the *C. elegans* beta-tubulin family comprises five more members, of which *tbb-1* and *tbb-2* are ubiquitously expressed and partially redundant, *ben-1/tbb-5* is broadly expressed in the nervous system, and *mec-7/tbb-3* and *tbb-4* are expressed only in touch receptor neurons [31]. The expressions of all three major beta-tubulins, *tbb-1*, *tbb-2*, and *ben-1*, were not changed in *lonp-1* mutant animals (Figure S1B), confirming a specific, yet unidentified, role of TBB-6 in mitochondrial dysfunction. However, the expression levels of the *tbb-6* gene and reporter were found to severely drop upon RNAi-mediated disruption of DLK-1 and SEK-3 or deletion of PMK-3 in both wt and *lonp-1* mutants (Figure 1D,E and Figure S2). Thus, we concluded that MAPK^mt^ signaling regulates both the basal and the *lonp-1*-induced expression of the *tbb-6* gene.

### 2.2. ATFS-1 Counteracts Activation of the MAPK^mt^ Pathway upon Lonp-1 Inactivation

**Figure 2:**
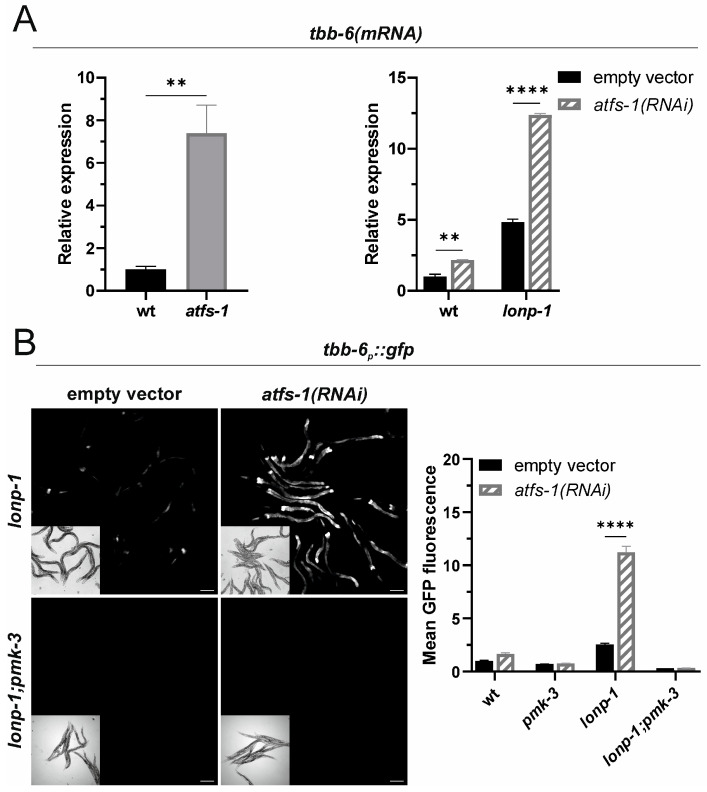
ATFS-1 inhibits activation of the MAPK^mt^ pathway upstream of PMK-3. (**A**) Relative mRNA quantification of endogenous *tbb-6* gene expression levels in wt and *atfs-1(gk3094)* loss-of-function mutants, or in wt and *lonp-1(ko)* animals subjected to RNAi against *atfs-1*. (**B**) Representative microscopy images and GFP fluorescence quantification of the *tbb-6_p_::gfp* reporter in wt, *lonp-1(ko)*, *pmk-3(ok169)* and *lonp-1(ko);pmk-3(ok169)* worms subjected to *atfs-1(RNAi)*. Experiments were performed on 1-day-old adult worms grown on NGM plates seeded with *E. coli* OP50 or with *E. coli* HT115 bacteria transformed with the indicated RNAi construct (and empty vector as a control) at 20 °C. Scale bar indicates 100 µm. Values are presented as means ± SEM, and asterisks denote statistical significance assessed with an unpaired Student’s *t*-test or with a two-way ANOVA followed by a post hoc Tukey’s test, ** *p* ≤ 0.01, **** *p* ≤ 0.0001.

Mitochondrial perturbation by LONP-1 deficiency has been demonstrated to elicit activation of several stress-responsive transcription factors, including SKN-1 and DAF-16, the worm orthologs of the mammalian NRF and Forkhead Box O (FOXO) factors, respectively [30]. Since the promoter of *tbb-6* contains putative binding sites for SKN-1 and DAF-16 transcription factors (WormBase version WS289), we tested their requirement for *tbb-6* induction in *lonp-1* mutants. RNAi targeting SKN-1 or DAF-16 did not reduce fluorescence of the *tbb-6_p_::gfp* reporter or *tbb-6 mRNA* levels (Figure S3), suggesting that these factors are not involved in the expression of *tbb-6* in *lonp-1* adults. Interestingly, genetic loss of *atfs-1* by the *gk3094* allele led to high *tbb-6* mRNA levels in an otherwise wt background (Figure 2A), and RNAi-induced depletion of *atfs-1* in a *lonp-1* background further increased the expression of the *tbb-6* gene or the *tbb-6_p_::gfp* reporter (Figure 2A,B).

These results support the notion that ATFS-1 counteracts the activity of the MAPK^mt^ pathway, as has been previously suggested for *isp-1(qm150)* mutants [24]. However, when two other UPR^mt^-associated factors, namely DVE-1 and UBL-5 [32], were knocked down by RNAi, no difference in the signal of the *tbb-6* reporter was detected (Figure S4), suggesting that ATFS-1 exerts a repressive function independently of the canonical UPR^mt^ signaling branch. The repressive function of ATFS-1 on *tbb-6* expression occurs upstream of PMK-3, as *atfs-1(RNAi)* was not able to increase the *tbb-6_p_::gfp* fluorescence in *lonp-1;pmk-3* double mutants (Figure 2B).

### 2.3. ATFS-1 Suppresses the Induction of cbp-3 in Lonp-1 Mutants

**Figure 3:**
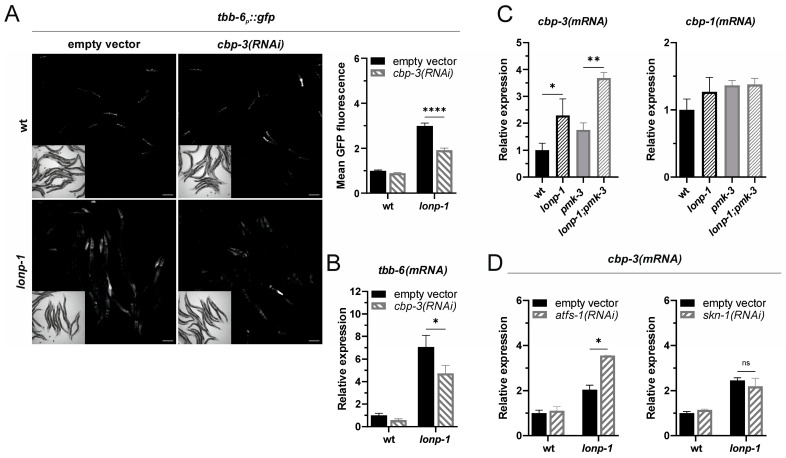
CBP-3 positively regulates MAPK^mt^ signaling and ATFS-1 suppresses *cbp-3* induction in *lonp-1* mutants. (**A**) Representative microscopy images and GFP fluorescence quantification of the *tbb-6_p_::gfp* reporter in wt and *lonp-1(ko)* worms subjected to RNAi against *cbp-3*. (**B**) Relative mRNA quantification of endogenous *tbb-6* gene expression levels in wt and *lonp-1(ko)* worms subjected to RNAi against *cbp-3*. (**C**) Relative mRNA quantification of *cbp-3* and *cbp-1* gene expression levels in wt, *lonp-1(ko)*, *pmk-3(ok169)* and *lonp-1(ko);pmk-3(ok169)* worms. (**D**) Relative mRNA quantification of *cbp-3* gene expression levels in wt and *lonp-1(ko)* animals subjected to RNAi against *atfs-1* or *skn-1*. Experiments were performed on 1-day-old adult worms grown on NGM plates seeded with *E. coli* OP50 or with *E. coli* HT115 bacteria transformed with the indicated RNAi construct (and empty vector as control) at 20 °C. Scale bar indicates 100 µm. Values are presented as means ± SEM, and asterisks denote statistical significance assessed with a two-way ANOVA followed by post hoc Tukey’s test, * *p* ≤ 0.05, ** *p* ≤ 0.01, **** *p* ≤ 0.0001.

It has been reported that *tbb-6* induction in *isp-1* mutant worms depends on *cbp-3*/*F40F12.7*, a homolog of the mammalian acetyltransferase CBP/p300 [24]. However, *cbp-3* is probably a pseudogene of *cbp-1*, bearing only the kinase-inducible domain interacting domain (KIX) and missing the histone acetyltransferase (HAT) domain of CBP-1 [33]. Thus, in contrast to *cbp-1(RNAi)*, we showed that RNAi against *cbp-3* does not cause developmental defects and arrest in worms, possibly due to lack of the HAT domain. When *lonp-1* mutant adults were subjected to *cbp-3(RNAi)*, the expression levels of *tbb-6_p_::gfp* or the endogenous *tbb-6* gene were significantly decreased (Figure 3A,B), supporting the role of *cbp-3* in the induction of *tbb-6* in a *lonp-1* background.

We further showed that the mRNA levels of *cbp-3*, and not those of *cbp-1*, were up-regulated in *lonp-1* mutants, independently of PMK-3 function (Figure 3C). Although the expression of *cbp-3* has been previously shown to increase upon induction of UPR^mt^ in an ATFS-1-dependent manner [25], we found that depletion of *atfs-1* enhances, rather than decreases, the expression of *cbp-3* in *lonp-1* mutants (Figure 3D), similarly to *tbb-6*. Depletion of SKN-1 had no effect on *cbp-3* transcript levels (Figure 3D), which is consistent with the lack of effects of *skn-1(RNAi)* in *tbb-6* mRNA levels. Thus, our data suggest that ATFS-1 suppresses the induction of *cbp-3* in *lonp-1* mutants and imply a role of the KIX domain of CBP-3 in the activation of the MAPK^mt^ pathway, placing it upstream of PMK-3/TBB-6 signaling.

### 2.4. Induction of the MAPK^mt^ Pathway in Lonp-1 Mutants Is Not MAD Pathway-Dependent

**Figure 4:**
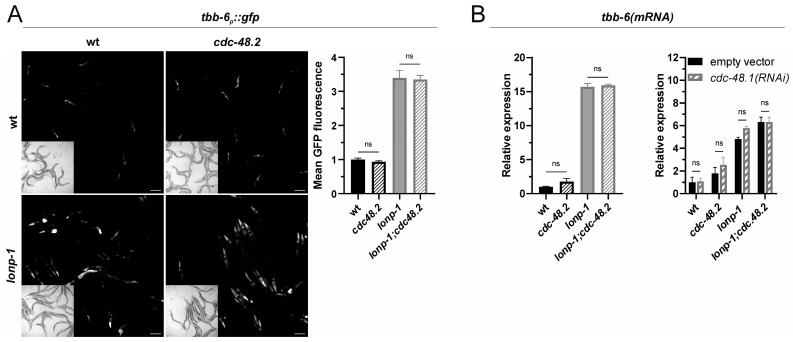
The MAD pathway does not mediate activation of the MAPK^mt^ pathway in *lonp-1* mutants. (**A**) Representative microscopy images and GFP fluorescence quantification of the *tbb-6_p_::gfp* reporter in 1-day adults of wt, *cdc-48.2(tm659)*, *lonp-1(ko)* and *lonp-1(ko);cdc-48.2(tm659)*, at 20 °C. (**B**) Relative mRNA quantification of endogenous *tbb-6* gene expression levels in 1-day adults of the aforementioned strains, grown on NGM plates seeded with *E. coli* OP50 or with *E. coli* HT115 bacteria transformed with the *cdc-48.1(RNAi)* construct (and empty vector as control), at 20 °C. Scale bar indicate 100 µm. Values were presented as mean ± SEM, and numeric data were analyzed with a two-way ANOVA followed by post hoc Tukey’s test, showing no significant (ns) differences.

Mitochondrial dysfunction in yeast strongly activates the mitochondria-associated degradation (MAD) pathway [34], and inhibition of MAD components could block *tbb-6_p_::gfp* induction in *isp-1* mutants [24]. The MAD pathway mediates ubiquitination and degradation of misfolded or damaged mitochondrial proteins under mitochondrial stress, analogously to the endoplasmic reticulum (ER)-associated degradation pathway (ERAD). Physical interaction of CBP-3 with proteins involved in protein ubiquitination, such as F57C12.2 [35,36] and *ubq-1* [37], has been reported; thus, we wondered whether CBP-3 functions with the MAD pathway to enable induction of MAPK^mt^ in *lonp-1* mutants. *C. elegans* has two orthologs of a highly conserved AAA^+^ ATPase, named p97/VCP/CDC48 in mammals, which direct ubiquitinated proteins from cellular membranes to the 26S proteasome for degradation [38,39,40]. The worm orthologs, CDC-48.1 and CDC-48.2, share 88% identity over the entire p97 protein, and both have essential and redundant functions [41]. We introduced a null mutation of *cdc-48.2* into *lonp-1* worms, but did not find a significant change in fluorescence of the reporter *tbb-6_p_::gfp* or the mRNA levels of *tbb-6* gene (Figure 4A). In a similar manner, depletion of *cdc-48.1* through RNAi, alone or in combination with *cdc-48.2(tm659)*, did not reduce the expression of *tbb-6* in *lonp-1* mutants (Figure 4B). These results indicate that the activity of the MAD pathway is not a prerequisite for MAPK^mt^ induction, at least in *lonp-1*-deficient worms, and *cbp-3* possibly influences PMK-3 activity through mechanisms independent of MAD signaling.

### 2.5. Loss of Lonp-1 Induces the ZIP-2 Innate Immune Response Effector

**Figure 5:**
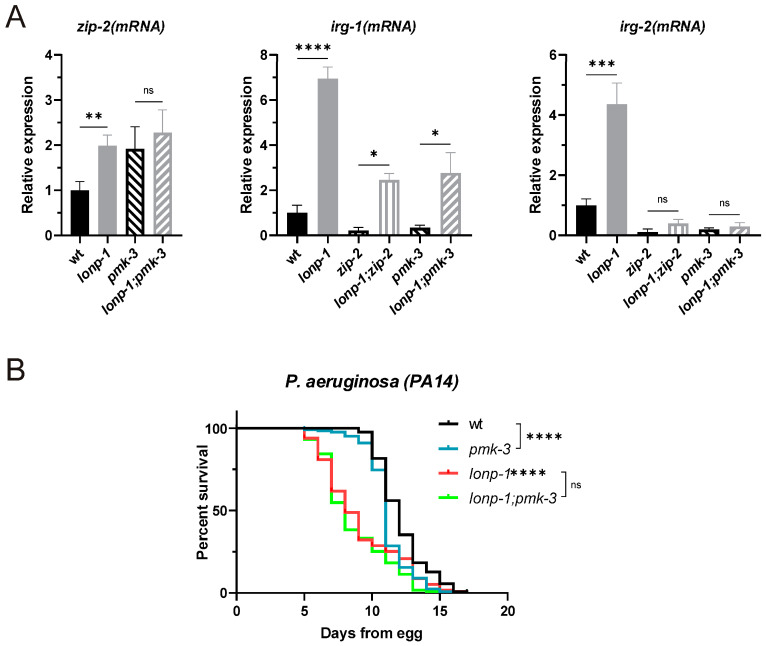
PMK-3-dependent activation of the bZIP transcription factor ZIP-2 does not confer resistance to pathogen infection. (**A**) Relative mRNA quantification of *zip-2*, *irg-1* and *irg-2* gene expression levels in 1-day-old adults of wt, *lonp-1(ko)*, *zip-2(ok3730)*, *lonp-1(ko);zip-2(ok3730)*, *pmk-3(ok169)* and *lonp-1(ko);pmk-3(ok169)* at 20 °C. Values are presented as means ± SEM and statistical significance was assessed with an unpaired Student’s *t*-test, * *p* ≤ 0.05, ** *p* ≤ 0.01, *** *p* ≤ 0.001 and **** *p* ≤ 0.0001. (**B**) Survival of slow killing Pseudomonas aeruginosa PA14 pathogen infection assay in 1-day-old adults of wt, *lonp-1(ko)*, *pmk-3(ok160)* and *lonp-1(ko);pmk-3(ok169)* on NGM plates seeded with *E. coli* OP50 in the presence of FUdR at 20 °C. Statistical significance was assessed via a Kaplan–Meier survival curve, **** *p* ≤ 0.0001.

Several studies of *C. elegans* have established that disruption of essential core processes, including mitochondrial function, can induce innate immune responses in worms [42,43]. In particular, UPR^mt^ has been shown to induce a subset of ATFS-1-dependent innate immunity genes, providing resistance to pathogenic bacteria *Pseudomonas aeruginosa*, independently of PMK-1/p38 MAPK [44]. One such gene is the infection response gene-1 (*irg-1*), a target of the bZIP transcription factor ZIP-2 [42,45,46], which was reported to be upregulated by ATFS-1 upon UPR^mt^ [25,44]. The expression of *irg-1* and *zip-2* was examined and was found to be significantly induced in *lonp-1* mutants compared to wt animals (Figure 5A). In addition to *irg-1*, the infection response gene-2 (*irg-2*), another ZIP-2 target [42,45], was upregulated upon *lonp-1* deletion (Figure 5A). Using a *zip-2(ok3730)* loss-of-function allele, we verified that ZIP-2 was required for basal expression of both *irg-1* and *irg-2*, whereas in a *lonp-1* mutant background, ZIP-2 was necessary only for *irg-2* induction (Figure 5A). Instead, *irg-1* could be upregulated in *lonp-1* worms by ZIP-2-independent mechanisms, as was previously reported for other conditions, e.g., suppression of translation [42].

Nevertheless, mitochondrial damage caused during infection by *P. aeruginosa* elicits a host defense, mediated in part by the ZIP-2 transcription factor, to promote organismal survival [47,48]. However, the increased activity of ATFS-1 and ZIP-2 in the *lonp-1* background was not sufficient to promote host resistance to infection by the *P. aeruginosa* strain PA14, and *lonp-1* adults were significantly susceptible to the pathogen compared to wt animals (Figure 5B). Given that ATFS-1 activity counteracts the activation of the MAPK^mt^ pathway, we tested whether genetic disruption of *pmk-3* could enhance the host defense response to PA14. As shown in Figure 5B, loss of *pmk-3* did not alter the hypersensitive phenotype of *lonp-1* mutants to pathogenic bacteria, although it had a negative impact on the immune response of wt worms. In spite of that, ZIP-2-dependent expression of *irg-1* and *irg-2* genes was compromised in both *pmk-3* and *lonp-1;pmk-3* mutants, similarly to *zip-2* deletion (Figure 5A). These results suggest a genetic interaction between PMK-3 and ZIP-2 when *lonp-1* is missing, which determines the activity of ZIP-2 and affects other functions than the innate immune response.

### 2.6. Disruption of the MAPK^mt^ Pathway Influences Lonp-1 Mutants’ Lifespan and Stress Responses

**Figure 6:**
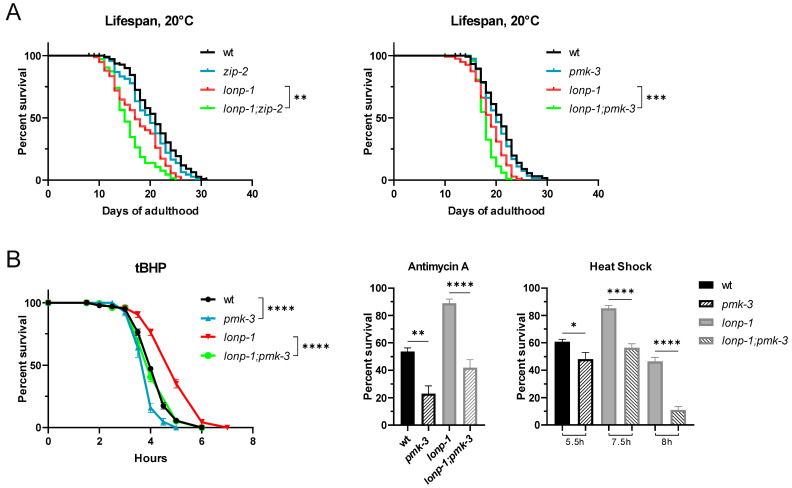
Loss of ZIP-2 or PMK-3 shortened the lifespan of *lonp-1* mutants and PMK-3 promotes organismal survival under stress. (**A**) Lifespan assays of wt, *lonp-1(ko)*, *zip-2(ok3730)*, *lonp-1(ko);zip-2(ok3730)*, *pmk-3(ok169)* and *lonp-1(ko);pmk-3(ok169)* worms on NGM plates seeded with *E. coli* OP50 at 20 °C. Replicates and statistical analysis of lifespan assays are shown in Table S3, ** *p* ≤ 0.01, *** *p* ≤ 0.001. (**B**) Survival of 1-day-old adults of wt, *lonp-1(ko)*, *pmk-3 ok160)* and *lonp-1(ko);pmk-3(ok160)* strains treated with tert-butyl hydroperoxide (tBHP, 10 mM), Antimycin A (40 μm for 24 h), or subjected to heat shock (at 35 °C for 5.5, 7.5 or 8 h). The percentage survival for all biological replicates was plotted, and an unpaired Student’s *t*-test was used to assess significance, * *p* ≤ 0.05, ** *p* ≤ 0.01 and **** *p* ≤ 0.0001.

Induction of the ZIP-2 pathway provides surveillance for several core cellular processes, and can also delay the ageing process by preserving mitochondrial homeostasis in aged worms [49]. Moreover, the extended lifespan of some, but not all, long-lived mitochondrial mutants was shown to require an active MAPK^mt^ pathway [24]. Considering the above genetic interaction between ZIP-2 and PMK-3, we examined the effects of disruption of each pathway on the lifespan of *lonp-1* mutants. Although worms deficient in the *lonp-1* gene display a shorter lifespan than the wt [30], deletion of either *zip-2* or *pmk-3* severely reduced the lifespan of *lonp-1* mutants, without affecting the normal lifespan of wt worms (Figure 6A and Table S3). This shortening of lifespans was not observed when *pmk-1*/p38 MAPK was deleted in the *lonp-1* mutant background, with loss of *pmk-1* to reduce only the brood size of both wt and *lonp-1* strains (Figure S5A,B and Table S3). Furthermore, deletion of *pmk-3*, *pmk-1* and *zip-2* (Figure S5C) did not have any impact on the growth of wt or *lonp-1* animals.

Worms bearing deficiency in the *lonp-1* gene are short-lived relative to the wt, but exhibit an increased resilience in some forms of exogenous stress, for instance, to specific oxidants or heat shock [30]. Here, we showed that, similarly to *pmk-1* deletion, loss of *pmk-3* renders both wt and *lonp-1* worms sensitive to oxidative stress induced by the organic peroxide tert-butyl hydroperoxide (tBHP) (Figure 6B and Figure S6A). Treatment of worms with antimycin, an inhibitor of complex III of the electron transport chain, revealed a general requirement for *pmk-3* to confront superoxide anion generation in mitochondria (Figure 6B), while *pmk-1* was required only in *lonp-1*-deficient animals (Figure S6A). These results suggest that PMK-3 is important for both wt and *lonp-1* adults to cope with oxidative stress. Moreover, deletion of *pmk-3* severely reduced the high survival rate of *lonp-1* mutants at 35 °C (Figure 6B), while loss of *pmk-1* had no effect on their extreme heat tolerance, with the double *lonp-1;pmk-1* mutants being similarly tolerant to single *lonp-1* mutants (Figure S6A). However, in a wt background, *pmk-1* deletion had a greater negative impact on thermotolerance compared to *pmk-3* deletion. Further studies are needed to address the key role of PMK-3 in the heat resistance of *lonp-1*, but it does not seem to involve *tbb-6* or *zip-2* functions (Figure S6B,C). Overall, our data support a central role of PMK-3/p38 MAPK in mediating the lifespan length and stress resistance under conditions of *lonp-1*-induced mitochondrial perturbations.

## 3. Discussion

In humans, mutations of the LonP1 gene are associated with a broad spectrum of diseases, including a rare multisystem developmental disorder known as CODAS (Cerebral, Ocular, Dental, Auricular, and Skeletal anomalies) syndrome, as well as neurodegeneration and cancer [27,28,29,50]. The mammalian protease LonP1 is a multifunctional enzyme with essential roles during development of cardiomyocyte and skeletal muscle cells [51,52]. Also, acting as both a chaperone and a protease, LonP1 plays a crucial role in protein quality control, preserving mitochondrial homeostasis in adults. An age-related decline in the expression and activity of LonP1 seems to contribute to structural and functional impairment of organs with age [29,51]. While genetic disruption of the LonP1 gene in mice and *Drosophila* results in embryonic lethality [53,54,55], *C. elegans lonp-1* knockout mutants are viable but exhibit disturbed mitochondrial and ROS homeostasis, resulting in impaired growth and a shortened lifespan [30]. *lonp-1*-deficient worms are extremely resistant to specific oxidants and heat stress due to induction of both mitochondrial and cytosolic stress signaling pathways. These include UPR^mt^, where increased activity of the ATFS-1 transcription factor was found to be essential for normal development of *lonp-1* mutants and determines the resistance of *lonp-1* adults to external oxidative stress [30]. Interestingly, lack of LONP-1 was recently reported to induce constitutive formation of mitochondrial stress granules, perhaps as an adaptive response [56]. Moreover, ATFS-1 activity in long-lived *isp-1* mitochondrial mutants could prevent the induction of another mitochondrial stress signaling pathway named MAPK^mt^, in which PMK-3/p38 MAPK upregulates the expression of the *tbb-6* beta-tubulin target gene in the intestines [24]. MAPKs are potent regulators of microtubule dynamics in mammals, and the DLK-1/PMK-3 pathway in *C. elegans* can regulate microtubule dynamics and endocytosis in neurons [57,58]. Although the exact role of TBB-6 in intestinal cells is still unknown, it has been suggested that it may function to enhance ADP entry into mitochondria during mitochondrial stress [24].

**Figure 7:**
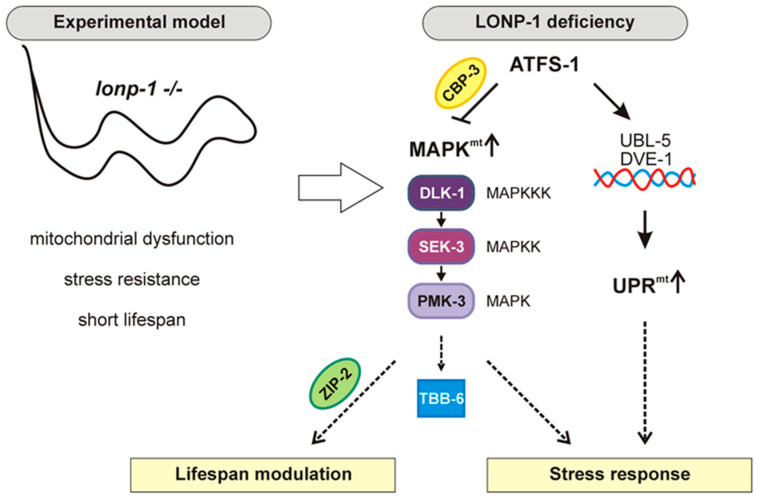
A schematic representation of the genetic interactions and molecular functions of MAPK^mt^ in worms deficient in the *lonp-1* gene.

Here, we demonstrated the induction of the MAPK^mt^ pathway in *lonp-1* mutants, which is consistent with the presence of a disturbed mitochondrial network and an accumulation of ROS in these animals. Based on our results discussed below, a schematic representation of the genetic interactions and molecular functions of MAPK^mt^ in worms deficient in the *lonp-1* gene is shown in Figure 7. Depletion of *atfs-1* further enhances the expression of the *tbb-6* marker, supporting the notion that ATFS-1 suppresses MAPK^mt^ activity, probably to fine-tune it. One regulator of ATFS-1 in PMK-3 activity may be the CBP ortholog CBP-3, which contains only the kinase-inducible domain interacting domain [33], and is required for *tbb-6* induction in *isp-1* mutants [24]. We found that *cbp-3* is upregulated in *lonp-1* worms and ATFS-1 suppresses this induction upstream of PMK-3/TTB-6 signaling. The combination of such protein interaction and phosphorylation events could be important for orchestrating the cellular processes and changing the metabolic status under two opposing signals in response to mitochondrial stress. For example, under conditions of moderate mitochondrial dysfunction, *C. elegans* can adapt and often exhibits an altered metabolism and an increased lifespan [59]. However, the ageing process can be accelerated when the activity of the different mitochondrial protein quality control systems is not in balance. In support of this, activity of the MAPK^mt^ pathway is required for the lifespan extension of some mitochondrial mutants [24]. We have shown that disruption of *atfs-1* in *lonp-1* adults reduced their lifespan [30] and here, deletion of *pmk-3* also shortened the lifespan of *lonp-1* animals. Moreover, our data as presented below uncovered a genetic interaction between PMK-3 and ZIP-2, which could influence the lifespan of *lonp-1* mutants.

ZIP-2 is a bZIP transcription factor with an established role in innate immunity [42,45,46]. Although mitochondrial dysfunction can induce immune responses in worms, mediated in part by ATFS-1 and ZIP-2 [44,47,48], the observed activation of ZIP-2 in *lonp-1* mutants was not sufficient to increase survival of mutants against pathogenic bacteria PA14. Moreover, the ZIP-2-dependent transcription of *irg-1* and *irg-2* target genes was abolished after depletion of *pmk-3*, signifying a requirement of the PMK-3 kinase for both basal and induced activity of the ZIP-2 factor, even though PMK-3 is not involved in innate immunity. Separate from its role in immunity, ZIP-2 is activated in response to age-associated mitochondrial dysfunction, independently of ATFS-1, and delays aging phenotypes by preserving mitochondrial homeostasis in aged worms [49]. In addition to this, ZIP-2 is activated and mediates lifespan extension under various dietary restriction conditions [60,61]. Since deletion of either *zip-2* or *pmk-3* significantly reduced the lifespan of *lonp-1* mutants, we speculate that this novel crosstalk between MAPK^mt^ and ZIP-2 is important in modulating the ageing process under mitochondrial dysfunction. This is strengthened by the observation that the DLK-1/PMK-3 signaling pathway declines with age, and this is overcome by mild mitochondrial stress [62]. Future studies investigating the underlying molecular mechanism and transcriptional targets are expected to shed light on the relevant ageing modulators.

We have previously shown that knockout of *lonp-1* induces a subset of stress-responsive genes associated with protective mechanisms, thereby safeguarding the mitochondrial and cytosolic proteome to defend the mitochondrial function [30]. Thus, here, we evaluated the consequences of *pmk-3* deletion on the resistance of *lonp-1* mutant adults to exogenous stresses. We confirmed that the activity of PMK-3 is indispensable for both oxidative and heat stress responses. More specifically, loss of PMK-3 significantly diminished the extreme heat resistance of *lonp-1* mutants, while deletion of *pmk-1* did not block this response. However, thermotolerance of wt animals was reduced when *pmk-1* was missing, which is consistent with a previous work, where heat was found to induce nuclear translocation of PMK-1 [23]. It has been suggested that PMK-1 might contribute to the elevated expression of *hsp* and other chaperone genes through hyperphosphorylation of heat shock factor I (HSF-1), analogously to the stress activation of mammalian HSF1 [63]. HSF-1 is the single worm homolog of the four mammalian HSFs, and its functions in thermal stress response are tightly regulated by post-translational modifications [64]. Given that the extreme thermotolerance of *lonp-1* mutants requires HSF-1 activity [30] and PMK-3, but not PMK-1 (this study), we propose that PMK-3 kinase mediates the function of HSF-1 or its regulators in *lonp-1* mutants in response to heat stress. These relationships might be evolutionarily conserved, as we have shown that genetic or pharmacological inhibition of human LonP1 in cancer cells induces the expression of genes involved in the heat shock response (HSR) [30]. Interestingly, new evidence exists for nuclear localization of mitochondrial LonP1 in mammalian cells, and this translocation is increased under heat stress, up-regulating HSF1 target genes [65]. Thus, it was suggested that LonP1 modulates the heat shock response through interaction with and possible degradation of HSF1 in the nucleus [65]. It is tempting to speculate that mammalian p38 MAPK could have a similar regulatory role with PMK-3 on LonP1 and HSF1 functions. Although in mammals there are four p38 isoforms that are highly similar to PMK-1 in worms, they exhibit differential expression/activation patterns and specific downstream effectors [1,66], raising the possibility that they may fulfill analogous cellular functions to the PMK-3 kinase in worms, promoting organismal fitness and stress responses.

## 4. Materials and Methods

### 4.1. C. elegans Strains and Culture Conditions

*C. elegans* strains were maintained at 20 °C on nematode growth medium (NGM) plates seeded with *Escherichia coli* OP50 as a food source. Whenever it was necessary, NGM plates with UV-killed bacteria supplemented with 40 μg/mL 5-fluoro-2′-deoxyuridine (FUdR; Sigma-Aldrich, St. Louis, MO, USA) were used to prevent progeny growth. All strains used in this study are listed in Table S1.

### 4.2. RNA Interference

RNAi experiments were conducted at 20 °C on NGM plates seeded with *E. coli* HT115 (DE3) bacteria, transformed with the indicated RNAi construct (primers listed in Table S2) or the appropriate empty vector (plasmid L4440 or T444T, Addgene, Watertown, MA, USA). Plates containing 2 mM isopropyl b-D-1-thiogalactopyranoside (IPTG; Applichem GmbH, Darmstadt, Germany) were incubated at 37 °C overnight to induce RNAi. Worms were synchronized from eggs on RNAi plates at 20 °C and transferred onto new RNAi plates when they reached L4 or the young prefertile adult stage (YA) for analysis. For post-developmental RNAi experiments, L4 to YA worms raised on OP50 plates were transferred onto RNAi plates for the indicated time.

### 4.3. Microscopic Analysis

Age-synchronized transgenic worms expressing the indicated fluorescence stress reporter were grown on OP50 or RNAi plates at 20 °C, and a microscopic analysis was performed in 1-day-old adults, considering the developmental delay in *lonp-1* mutants. Worms were immobilized with levamisole (Sigma-Aldrich, St Louis, MO, USA) and mounted on 2% agarose pads on glass microscope slides. Images of worms were captured via fluorescent microscopy using a Leica DMRA upright fluorescent microscope equipped with a Hamamatsu ORCA-flash 4.0 camera with a 10× or 40× objective lens. All strains were assayed in parallel and microscopy settings were kept the same in each experiment. Fluorescence was measured as the average pixel intensity with ImageJ 1.52p (Fiji) [67] in the whole captured worm image or in standardized regions of the intestine. At least 50 worms per strain and condition were measured in three biological replicates, and the mean of calculated values was plotted as the mean fluorescence intensity ± SEM. Representative fluorescence and bright field micrographs are shown for each genotype and condition.

### 4.4. Growth Rate and Fecundity Assays

Worms were synchronized by placing 10 gravid adults of each strain on NGM plates with adequate food (OP50) and they were allowed to lay eggs for 2–3 h before removing. Eggs were left to hatch at 20 °C, and the number of progenies in each developmental stage after 68 h was scored. The total brood size was determined at 20 °C by picking 10 L4 stage nematodes from an NGM plate and placing one nematode onto separate individual NGM plates with OP50. Individual animals were transferred onto new plates daily until the end of the spawning period of worms. The total number of F1 progeny hatched in each plate for each individual was counted.

### 4.5. Lifespan Analysis

Animals were synchronized by egg-laying, and 100–150 healthy L4 larvae of each strain, were transferred to three NGM plates (approximately 40 per plate) seeded with OP50 bacteria, at 20 °C. Animals were moved to fresh plates every 2–4 days and survival was scored daily by gentle prodding. Worms that failed to respond were considered dead, whereas worms raptured, burrowed into the agar, bagged (that displayed internal hatching of eggs), or animals that crawled off the plates were censored in the analysis. Statistical analysis was performed by comparing each population to the appropriate control and *p*- values were determined using the log-rank (Mantel-Cox) test. Replicates were carried out as indicated in Table S3.

### 4.6. Stress Sensitivity Assays

In all assays, animals were synchronized by egg laying on NGM plates with OP50 bacteria at 20 °C. Heat shock assays were performed by shifting 1-day-old adults onto three fresh plates containing approximately 30 animals per plate from 20 °C to 35 °C for the indicated time points. The percentage of alive animals was scored following 16 h of recovery at 20 °C by assessing the response to gentle touch. For the tBHP-induced oxidative stress assay, 1-day-old adult worms were placed on freshly prepared tBHP plates [30] containing 10 mM tBHP (from a stock solution of 70% in water, Sigma-Aldrich, St Louis, MO, USA) devoid of bacteria. During the initial two hours, worms were periodically relocated to the center of the plate, as they tried to “escape” from the plate and avoid tBHP, and survival was counted every hour. For antimycin A (Sigma-Aldrich, St Louis, MO, USA)-induced oxidative stress, synchronized L4 larvae of each strain were placed on plates containing 40 μM FUdR for 24 h. The next day, approximately 30 worms per plate were transferred onto fresh plates without FUdR seeded with UV-killed bacteria containing 40 μM antimycin A. Survival was monitored after 24 h on three plates per strain. At least three independent experiments were performed for each stress assay. Resilience against the human opportunistic pathogen *P. aeruginosa* was tested using the pathogen infection assay, performed by SunyBiotech (https://www.sunybiotech.com, accessed on 1 February 2023). In brief, age-synchronized worms of the indicated genotype were grown at 20 °C on NGM plates seeded with OP50 bacteria. Approximately 80 of the synchronized young adults of each strain were transferred to fresh FUdR-containing plates seeded with the *P. aeruginosa* strain PA14. Viability was scored daily and *p*-values were determined using the log-rank (Mantel-Cox) test.

### 4.7. RNA Extraction and Quantitative Reverse Transcription PCR (qRT-PCR)

Total RNA was extracted from frozen worm pellets, each containing 200–300 worms, as described previously [30]. In each experimental set, a minimum of three biological replicate samples were collected and analyzed independently. Relative mRNA quantities were determined using the comparative Ct method for quantification, with each sample independently normalized to the endogenous reference gene (*ama-1*). Gene expression data are presented as the average fold change of all biological replicates relative to the control. The primer sequences used for qRT-PCR are shown in Table S2.

### 4.8. Statistics

Graphs were constructed and statistical analyses were performed in GraphPad Prism version 8.0.0 for Windows (GraphPad Software, San Diego, CA, USA, www.graphpad.com/scientificsoftware/prism, accessed on 10 December 2021). Statistical analyses were performed by comparing each sample to the appropriate control in the same condition and *p*-values were determined by a Student’s *t*-test and depicted as follows: **** *p* < 0.0001; *** *p* = 0.0001–0.001; ** *p* = 0.001–0.01; * *p* = 0.01–0.05; ns indicates not significant with a *p*-value ≥ 0.05. For analyses involving multiple strains and conditions, a two-way ANOVA with a Tukey’s multiple comparisons analysis was used to assess the significance and differences between groups.

## 5. Conclusions

In *C. elegans*, the *lonp-1* gene encodes a mitochondrial AAA^+^ protease, which has been shown to play a crucial role in protein quality control across species. In this work, we found that worms deficient in *lonp-1* have an activated mitochondrial PMK-3/p38 MAPK signaling pathway. In *lonp-1* mutants, the enhanced activity of the ATFS-1 transcription factor counteracted activation of the MAPK^mt^ pathway, pointing to the existence of interconnected and fine-tuned defense mechanisms in response to mitochondrial stress. In support of this notion, both ATFS-1 and PMK-3 activities positively regulated the *lonp-1* mutants’ lifespan and oxidative stress response. In addition to this, we demonstrated that PMK-3 was required for both the basic and *lonp-1*-induced transcriptional activity of ZIP-2, a bZIP transcription factor that has previously been shown to preserve mitochondrial homeostasis in aged worms independently of innate immunity. Since loss of ZIP-2 activity reduced the lifespan of *lonp-1* animals, we suggest that this genetic interaction between PMK-3 and ZIP-2 can influence the ageing process in the face of mitochondrial damage. Finally, the extreme thermotolerance of *lonp-1* mutants was found to require PMK-3 activity, uncovering a new role of MAPK^mt^ in the heat stress response when LONP-1 is missing.

## Data Availability

Data are contained within the article and Supplementary Materials.

## Supplementary Materials

The following supporting information can be downloaded at: https://www.mdpi.com/article/10.3390/ijms242417209/s1.

## Abbreviations

MAPK	Mitogen-Activated Protein Kinase
MAPKK	Mitogen-Activated Protein Kinase Kinase
MAP3K	Mitogen-Activated Protein Kinase Kinase Kinase
MAPK^mt^	Mitochondrial MAPK
UPR^mt^	Mitochondrial Unfolded Protein Response
UPR^cyt^	Cytoplasmic Unfolded Protein Response
ATF	Activating Transcription Factor
SARM	Sterile α and Armadillo Motif
ASK1	Apoptosis Signal-Regulating Kinase 1
MKK	Mitogen-Activated Protein Kinase Kinase
NRF	Nuclear Factor-Erythroid 2-Related Factor
FOXO	Forkhead Box O
CBP	CREB-Binding Protein
MAD	Mitochondrial-Associated Degradation
bZIP	Basic Leucine Zipper
RNAi	RNA Interference
tBHP	Tert-Butyl Hydroperoxide
hsp	Heat Shock Protein
HSF	Heat Shock Factor
HSR	Heat Shock Response
