# Supplementary material for: Mitochondrial p38 Mitogen-Activated Protein Kinase: Insights into Its Regulation of and Role in LONP1-Deficient Nematodes
Source: Int J Mol Sci. 2023 Dec 7;24(24):17209. doi: 10.3390/ijms242417209 (PMC10743222; doi:10.3390/ijms242417209)
Supplement: Supplementary file 1 [file ijms-24-17209-s001.zip › Supplementary Figures.pdf]

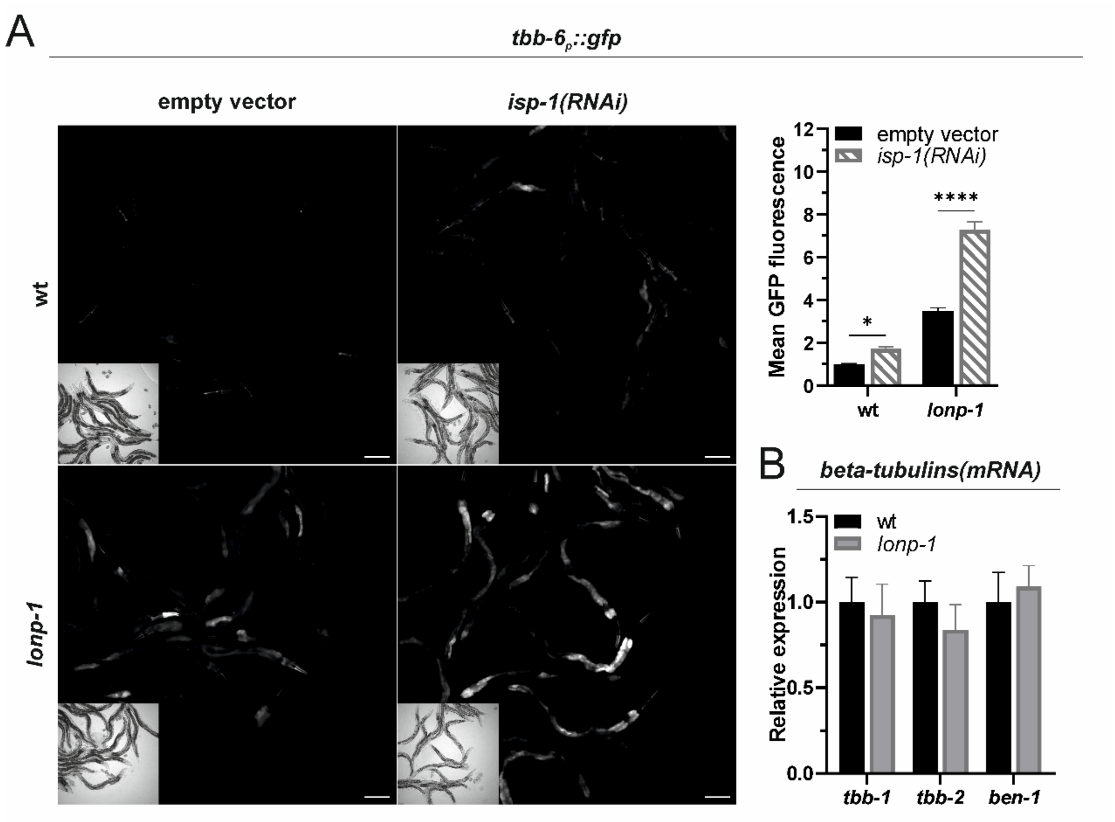

Supplementary Figure S1. Silencing of *isp-1* augments the MAPK^{mt} pathway induction in *lonp-1* mutants, whereas the expression of the three major beta-tubulins is not affected by *lonp-1* deletion. (**A**) Representative microscopy images and GFP fluorescence quantification of the *tbb-6*$_p$*::gfp* reporter in 1-day adults of wt and *lonp-1(ko)* strains, fed with *isp-1(RNAi)* bacteria, at 20°C. Scale bar indicates 100 μm. Values were presented as mean ± SEM, and asterisks denote statistical significance assessed with a two-way ANOVA followed by post hoc Tukey's test, *$p \leq 0.05$ and ****$p \leq 0.0001$. (**B**) Relative mRNA quantification of *tbb-1, tbb-2* and *ben-1* genes expression levels in 1-day adults of wt and *lonp-1(ko)* strains, raised on NGM plates with OP50 bacteria, at 20°C. Values were presented as mean ± SEM, and significance was assessed with unpaired Student's *t*-test.

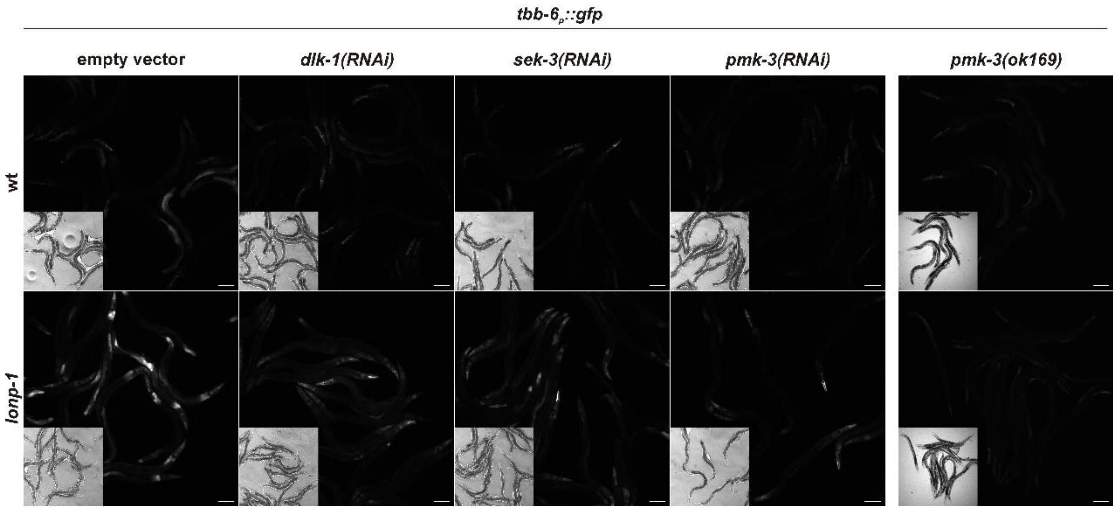

Supplementary Figure S2. The induction of the *tbb-6* reporter gene in *lonp-1* mutants is dependent on the DLK-1/SEK-3/PMK-3 signaling cascade. Representative microscopy images and GFP fluorescence of the *tbb-6*$_p$*::gfp* reporter in 1-day adults of wt and *lonp-1(ko)* strains, at 20°C. Worms were grown on NGM plates seeded with *E. coli* HT115 bacteria transformed with the indicated RNAi construct or empty vector as control. Scale bar indicates 100 µm.

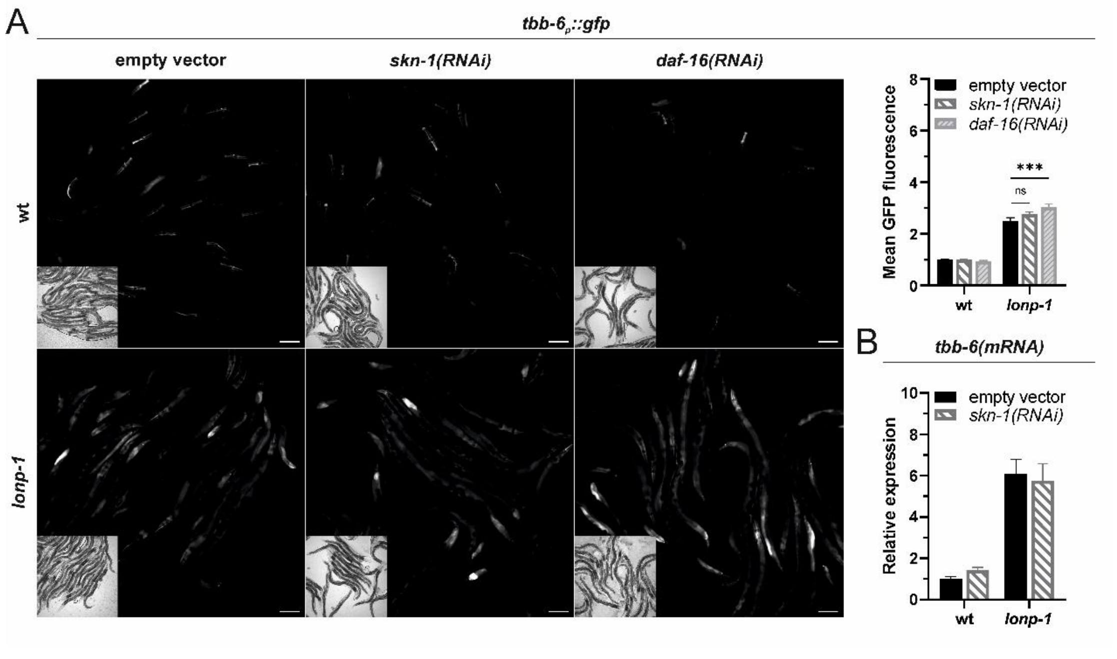

Supplementary Figure S3.

The SKN-1/NRF and DAF-16/FOXO stress-responsive transcription factors are not responsible for the induction of *tbb-6* in *lonp-1* mutants. (**A**) Representative microscopy images and GFP fluorescence quantification of the *tbb-6*$_p$*::gfp* reporter in 1-day adults of wt and *lonp-1(ko)* worms, fed with *skn-1(RNAi)* or *daf-16(RNAi)* bacteria. Scale bar indicate 100 µm. Values were presented as mean ± SEM, and significance was assessed with a two-way ANOVA followed by post hoc Tukey's test, ***$p \leq 0.001$. (**B**) Relative mRNA quantification of endogenous *tbb-6*

gene expression levels in 1-day adults of wt and *lonp-1(ko)* worms, fed with *skn-1(RNAi)* bacteria, at 20°C. Values were presented as mean ± SEM, and significance was assessed with unpaired Student's *t*-test.

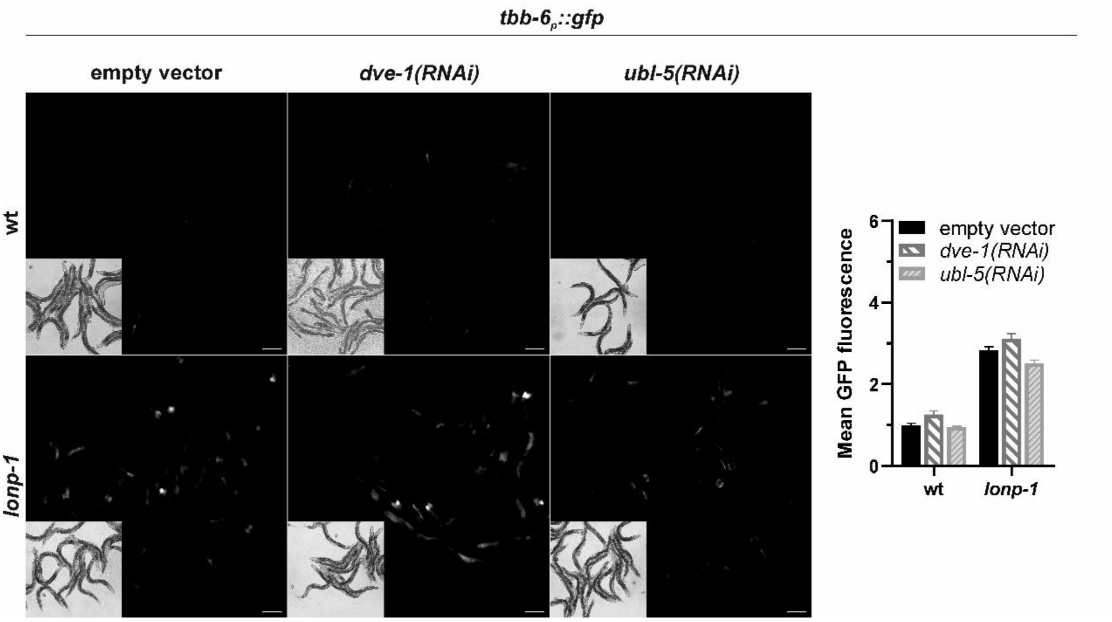

Supplementary Figure S4. Depletion of the DVE-1 and UBL-5 UPR^{mt}-associated factors do not suppress *tbb-6* induction in *lonp-1* mutants. Representative microscopy images and GFP fluorescence quantification of the *tbb-6$_p$::gfp* reporter in 1-day adults of wt and *lonp-1(ko)* worms, at 20°C. Worms were grown on NGM plates seeded with *E. coli* HT115 bacteria transformed with the indicated RNAi construct. Scale bar indicates 100 µm. Values are represented as mean ± SEM, and significance was assessed with a two-way ANOVA followed by post hoc Tukey's test.

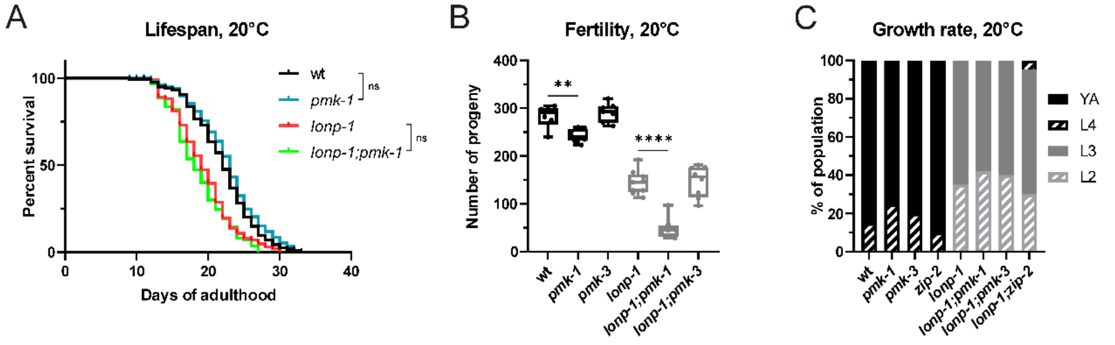

Supplementary Figure S5. Loss of *pmk-1* does not influence the lifespan or growth rate but reduces the brood size of both wt and *lonp-1* worms. (**A**) Lifespan assays of wt, *lonp-1(ko), pmk-1(km25)* and *lonp-1(ko);pmk-1(km25)* worms. Replicates and statistical analysis of lifespan assays are shown in Supplementary Table S3. (**B**) Fertility assay of wt, *lonp-1(ko), pmk-1(km25), lonp-1(ko);pmk-1(km25), pmk-3(ok169)* and *lonp-1(ko);pmk-3(ok169)* worms. Plot shows the mean number of viable progenies per individual in all biological replicates.

Unpaired Student's *t*-test was used to assess significance, $**p \leq 0.01$ and $***p \leq 0.001$. (**C**) Growth rate of wt, *lonp-1(ko), pmk-1(km25)*, *lonp-1(ko);pmk-1(km25), pmk-3(ok169)*, *lonp-1(ko);pmk-3(ok169)*, *zip-2(ok3730) and lonp-1(ko);zip-2(ok3730)* worms, showing the percentage of population in each developmental stage (Larval stages L2-L4 and Young Adult) ~68 hours post-egg-laying at 20°C.

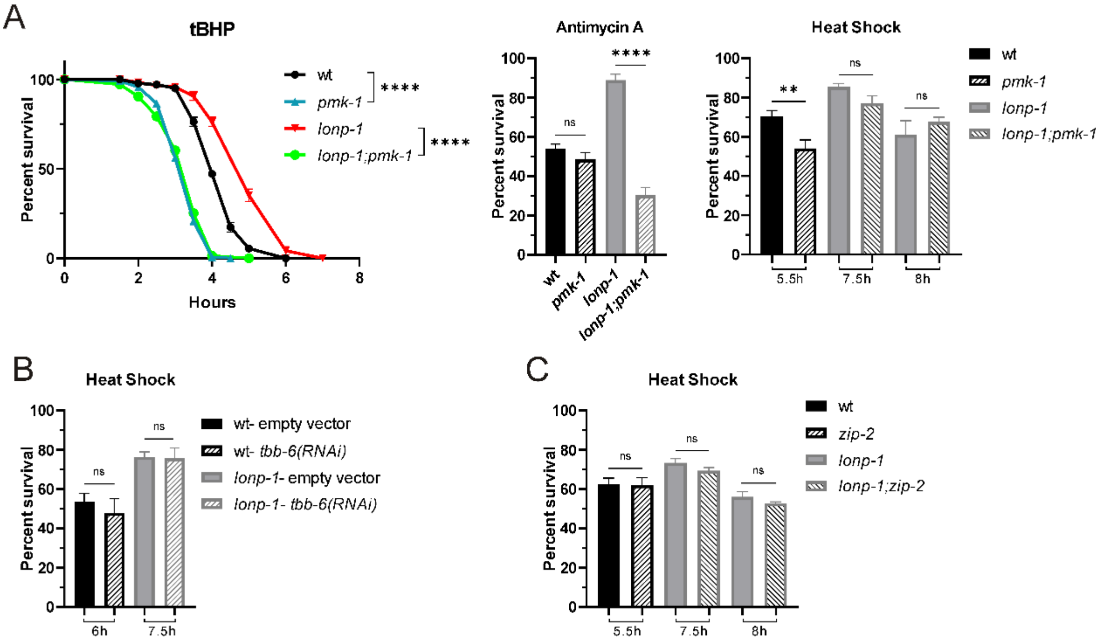

Supplementary Figure S6. PMK-1 is required for oxidative stress resistance but not for thermotolerance of lonp-1 mutants, but *tbb-6* and *zip-2* are not involved in heat resistance. (**A**) Survival of wt, *lonp-1(ko), pmk-1(km25)* and *lonp-1(ko);pmk-1(km25)* on tBHP (10 mM), Antimycin A (40 µm for 24 hours) or upon heat-shock (at 35°C for 5.5, 7.5 and 8 hours). (**B**) Survival of wt and *lonp-1(ko)* worms subjected to *tbb-6(RNAi)* upon heat-shock (at 35°C for 6 and 7 hours). (**C**) Survival of wt, *lonp-1(ko), zip-2(ok3730) and lonp-1(ko);zip-2(ok3730)* upon heat-shock (at 35°C for 5.5, 7.5 and 8 hours). The percentage survival for all biological replicates was plotted. Values are represented as mean ± SEM, and significance was assessed with unpaired Student's *t*-test, $**p \leq 0.01$, $***p \leq 0.001$ and $****p \leq 0.0001$.
