# Supplementary material for: Mitochondrial p38 Mitogen-Activated Protein Kinase: Insights into Its Regulation of and Role in LONP1-Deficient Nematodes
Source: Int J Mol Sci. 2023 Dec 7;24(24):17209. doi: 10.3390/ijms242417209 (PMC10743222; doi:10.3390/ijms242417209)
Supplement: Supplementary file 1 [file ijms-24-17209-s001.zip › Table S1.pdf]

Supplementary Table S1. List of *C. elegans* strains used in this study

Strain	Genotype	Information	Reference
N2	N2 (Bristol)	Wild-type (wt)	CGC
BRF791	*lonp-1(ko)I*	CRISPR/Cas9-mediated knockout of *lonp-1*	*Taouktsi et al., 2022*
BS3383	*pmk-3(ok169) IV*	Loss-of-function; ~1.5kb deletion	CGC
BRF854	*lonp-1(ko);pmk-3(ok169) I;IV*	Cross of BRF791 males with BS3383 hermaphrodites	This study
KU25	*pmk-1(km25)IV*	Loss-of-function; 375bp deletion	CGC
BRF864	*lonp-1(ko);pmk-1(km25) I;IV*	Cross of BRF791 males with KU25 hermaphrodites	This study
FX659	*cdc-48.2(tm659) II*	Loss-of-function; 639bp deletion	CGC
BRF860	*lonp-1(ko);cdc-48.2(tm659) I;II*	Cross of BRF791 males with BRF867 hermaphrodites	This study
VC3201	*atfs-1(gk3094) V*	Loss-of-function; 881bp deletion	CGC
VC3056	*zip-2(ok3730) III*	Loss-of-function; 476bp deletion	CGC
BRF843	*lonp-1(ko);zip-2(ok3730) I;III*	Cross of BRF791 males with VC3056 hermaphrodites	This study
SLR115	*dvIs67[tbb-6p::gfp + myo-3p::dsRed] N/A*	Used as MAPK^{mt} marker (*Munkacsy et al., 2016*)	CGC
BRF859	*lonp-1(ko); dvIs67 (tbb-6p::GFP+myo-3p::dsRed)*	Cross of BRF791 males with SLR115 hermaphrodites	This study
BRF874	*pmk-3(ok169); dvIs67 (tbb-6p::GFP+myo-3p::dsRed)*	Cross of SLR115 males with BRF854 hermaphrodites	This study
BRF875	*pmk-3(ok169); lonp-1(ko); dvIs67 (tbb-6p::GFP+myo-3p::dsRed)*	Cross of SLR115 males with BRF854 hermaphrodites	This study
BRF872	*pmk-1(km25); dvIs67 (tbb-6p::GFP+myo-3p::dsRed)*	Cross of SLR115 males with BRF864 hermaphrodites	This study

**Strain**	**Genotype**	**Information**	**Reference**
BRF873	*pmk-1(km25); lonp-1(ko); dvIs67 (tbb-6p::GFP+myo-3p::dsRed)*	Cross of SLR115 males with BRF864 hermaphrodites	This study
BRF862	*cdc-48.2(tm659);dvIs67 (tbb-6p::GFP+myo-3p::dsRed)*	Cross of SLR115 males with BRF860 hermaphrodites	This study
BRF868	*lonp-1(ko);cdc-48.2(tm659);dvIs67 (tbb-6p::GFP+myo-3p::dsRed)*	Cross of SLR115 males with BRF860 hermaphrodites	This study
SJ4100	*zcIs13[hsp-6$_p$::gfp, lin-15(+)]V*	Used as UPR^{mt} marker (*Yoneda et al., 2004*)	CGC
BRF789	*lonp-1(ko)I*; zcIs13(*hsp-6$_p$::gfp, lin-15(+)]V*	Cross of BRF791 males with SJ4100 hermaphrodites	*Taouktsi et al., 2022*
