# Supplementary material for: Mitochondrial p38 Mitogen-Activated Protein Kinase: Insights into Its Regulation of and Role in LONP1-Deficient Nematodes
Source: Int J Mol Sci. 2023 Dec 7;24(24):17209. doi: 10.3390/ijms242417209 (PMC10743222; doi:10.3390/ijms242417209)
Supplement: Supplementary file 1 [file ijms-24-17209-s001.zip › Table S2.pdf]

**Supplementary Table S2. List of primers used in this study**

| Primer name | Oligonucleotide sequences (5´→3´) | Target |
|---|---|---|
| Primers for tracking *C. elegans* strains | | |
| CD.Cas9.LON.Frw | ACGTTTTACTCGGGATCTGGT | *lonp-1(ko)* by CRISPR/Cas9 |
| CD.Cas9.LON.Rev | ATCGACAAGCTTCAAACCGC | |
| Lon Del Frw | CGTTTTACTCGGGATCTGGTC | *lonp-1(ko)* mutants |
| Lon Del Rev | GCTTCCACAGCAGATTCCC | |
| Lon wt Frw | TTAACCGGTCCATGTCACCGATTCAAGTGCC | |
| Lon wt Rev | GATATTACCGAATCGTCTGCAC | |
| pmk-1 Frw | GTTGCCATGACCTCAGAGC | *pmk-1(km25)* mutants |
| pmk-1 Rev | CAGTTTGACGTGCCAATCCA | |
| pmk-3 Frw | TTTTCACTGCGTCTCAATCG | *pmk-3(ok169)* mutants |
| pmk-3 Rev | GCATTACATAGCCAGTTTCC | |
| cdc-48.2 Frw | TCTATTGATGGAGCCCTTCG | *cdc-48.2(tm659)* mutants |
| cdc-48.2 Rev | CACGAGCACTTCTGTCCTGA | |
| atfs-1 Frw | ATGTTTTCCCGTGTGGGACGT | *atfs-1(gk3094)* mutants |
| atfs-1 Rev | CGAACATTTTTCCGTGAAGATAACT | |
| zip-2 Frw | ACTCACCGCTCCAGGATG | *zip-2(ok3730)* mutants |
| zip-2 Del | TGAGGTTGGTGAATAAGGGA | |
| Primers for RNAi constructs | | |
| atfs-1i Frw | ATGTTTTCCCGTGTGGGACGT | *atfs-1(RNAi)* |
| atfs-1i Rev | CGAACATTTTTCCGTGAAGATAACT | |
| cbp-1i Frw | AACTGCAGCTAATTGATGGATGAACCACCATC | *cbp-1(RNAi)* |
| cbp-1i Rev | GCTCTAGACCCTTGATGCATCATTGGATATC | |

| | | |
|---|---|---|
| cbp-3i Frw | GAATCAATCATGGAACAACTGTG | *cbp-3(RNAi)* |
| cbp-3i Rev | CACACAAATGTTCTCAACGTGAG | |
| cdc-48.1i Frw | ATGGCCTCGGTTCCAACGCA | *cdc-48.1(RNAi)* |
| cdc-48.1i Rev | AACTGCAGCCACAAGGTTAGAACGACCC | |
| daf-16i Frw | CAATGAGATTTATCAATGGTTCTC | *daf-16α(RNAi)* |
| daf-16i Rev | CGATTGAGTTCGGGGACTG | |
| dlk-1i Frw | ATCTAGACGCTATCTCCGAACTTGAA | *dlk-1(RNAi)* |
| dlk-1i Rev | ACTCGAGAAAATGATTGCCATCTGAGC | |
| dve-1i Frw | ATCTAGACACTCTTTACGAAATTCCACG | *dve-1(RNAi)* |
| dve-1i Rev | ACTCGAGTGGGTCGAACATCAGG | |
| isp-1i Frw | AGCTCTGCAGGCATCAACTGGGGCAATGGC | *isp-1(RNAi)* |
| isp-1i Rev | CGGACTCGAGGATAGAACTCCTCCAGCTGT | |
| pmk-1i Frw | GCTCTAGATGTTTCCACAGACAAC | *pmk-1(RNAi)* |
| pmk-1i Rev | CCGCTCGAGATCAGATCCAGGGAAC | |
| pmk-3i Frw | TCGAGATGTATACGTTGTCACTG | *pmk-3(RNAi)* |
| pmk-3i Rev | GATTAACGGTGATGAGCTGAG | |
| sek-3i Frw | TTCTAGAAATCGACACAATCTGGC | *sek-3(RNAi)* |
| sek-3i Rev | ACTCGAGCCGACATCAACAGACTGAG | |
| skn-1i Frw | GGGAAGCTTCCAACTACGCCTACATTGG | *skn-1(RNAi)* |
| skn-1i Rev | GGGCTCGAGCTTGTCGTGACGATCCGTG | |
| ubl-5i Frw | TTTGAAGAAGTTGATCGCTGC | *ubl-5(RNAi)* |
| ubl-5i Rev | TGAATCCCTCGTGAATCTCG | |
| Primers for RT-qPCR | | |
| ben-1 RT1 | CGTATGTCAATGAGAGAAGTCG | *ben-1* |
| ben-1 RT2 | CGGCGGAACATTGCTGTAAAT | |

| | | |
|---|---|---|
| cbp-1 RT1 | TCCTCCGAATGGACAAGTTC | *cbp-1* |
| cbp-1 RT2 | CAATAGCCGTTGGTGGTCTT | |
| cbp-3 RT1 | GAATCAATCATGGAACAACTGTG | *cbp-3* |
| cbp-3 RT2 | GGACATCAGTCAAGAACCAAC | |
| irg-1 RT1 | AATATTTCATGTACAATAAGGCGTTG | *irg-1* |
| irg-1 RT2 | CCTTTGATTTTTGAGACCATAATTTC | |
| irg-2 RT2 | GCTCGAGACTAGATCCCAAGTG | *irg-2* |
| irg-2 RT2 | CGACGAGTTTTACTTCCGAAAA | |
| irg-5 RT1 | GCAAATAACGATCAGGTTGCAT | *irg-5* |
| irg-5 RT2 | AAGCGGTGTAATCAGGTCCA | |
| pmk-3 RT1 | TCGAGATGTATACGTTGTCACTG | *pmk-3* |
| pmk-3 RT2 | GCGAGATTTCCTGGTTTCAAG | |
| tbb-1 RT1 | GCATGTCCATGAGAGAGGTTG | *tbb-1* |
| tbb-1 RT2 | AGCTCCTGGATAGCAGTGGA | |
| tbb-2 RT1 | GACGCATGTCGATGAGAGAG | *tbb-2* |
| tbb-2 RT2 | GGCGGAACATAGCAGTGAAC | |
| tbb-6 RT1 | ACGTTCAAGTTGGACAATGTGG | *tbb-6* |
| tbb-6 RT2 | GAACATACTTTCGACCTTCCATC | |
| zip-2/RT1 | CCAAGTATGGAGCCGCTATC | *zip-2* |
| zip-2/RT2 | GGCAGGTTTCTGCAAGAGTT | |
