# Supplementary material for: Mitochondrial p38 Mitogen-Activated Protein Kinase: Insights into Its Regulation of and Role in LONP1-Deficient Nematodes
Source: Int J Mol Sci. 2023 Dec 7;24(24):17209. doi: 10.3390/ijms242417209 (PMC10743222; doi:10.3390/ijms242417209)
Supplement: Supplementary file 1 [file ijms-24-17209-s001.zip › Table S3.pdf]

**Supplementary Table S3. Analysis of lifespan assay results**

| Strain | Genotype | Median survival | Temperature | Mean/SEM | n(T/C)[a] | *p*-value vs wt strain | *p*-value vs *lonp-1(ko)* |
|---|---|---|---|---|---|---|---|
| N2 | wt | 21 | 20ºC | 20.67±0.333 | 138/10 | - | - |
| BRF791 | *lonp-1(ko)* | 19 | | 18.67±0.333 | 121/14 | <0.0001(****) | - |
| BRF852 | *pmk-3(ok169)* | 20 | | 20.33±0.333 | 124/6 | 0.3319 (ns) | - |
| BRF851 | *lonp-1(ko);pmk-3(ok169)* | 18 | | 17.67±0.333 | 126/25 | - | 0.0005(***) |
| N2 | wt | 22 | 20ºC | 22.33±0.666 | 126/12 | - | - |
| BRF791 | *lonp-1(ko)* | 19 | | 19.67±0.666 | 125/20 | <0.0001(****) | - |
| BRF564 | *pmk-1(km25)* | 23 | | 23.33±0.666 | 108/15 | 0.2181(ns) | - |
| BRF864 | *lonp-1(ko);pmk-1(km25)* | 18 | | 18.17±0.166 | 122/11 | - | 0.1698(ns) |
| BRF791 | *lonp-1(ko)* | 19 | 20ºC | 19.33±0.333 | 128/9 | | |
| BRF851 | *lonp-1(ko);pmk-3(ok169)* | 18 | | 18.33±0.333 | 106/20 | | 0.0084(**) |
| BRF864 | *lonp-1(ko);pmk-1(km25)* | 19 | | 19.00±0.000 | 110/9 | | 0.0701(ns) |
| N2 | wt | 21 | 20ºC | 20.67±0.333 | 120/12 | | |
| BRF791 | *lonp-1(ko)* | 17 | | 17.33±0.333 | 86/16 | <0.0001(****) | |
| VC3056 | *zip-2(ok3730)* | 20 | | 20.13±0.314 | 146/36 | 0.0909(ns) | |
| BRF843 | *lonp-1(ko);zip-2(ok3730)* | 15 | | 15.25±0.478 | 162/31 | - | 0.0034(**) |

[a]: n=Number of animals of each strain, counted in at least three plates (T=Total, C=Censored animals)
